# Dermatomyosite et cancer rectal: à propos d’un cas avec revue de la littérature

**DOI:** 10.11604/pamj.2019.33.122.14509

**Published:** 2019-06-18

**Authors:** Hajar Ouahbi, Meriem Benhami, Lamiae Nouikh, Nissrine Acharfi, Awatef Kelati, Karima Oualla, Zineb Benbrahim, Fatima Zahra Elmrabet, Samia Arifi, Fatimazohra Mernissi, Nawfel Mellas

**Affiliations:** 1 Service d’Oncologie Médicale, CHU Hassan II, Fès, Maroc; 2 Service de Dermatologie, CHU Hassan II, Fès, Maroc

**Keywords:** Dermatomyosite, cancer rectal, anatomopathologie, chimiothérapie, Dermatomyositis, rectal cancer, anatomopathology, chemotherapy

## Abstract

La dermatomyosite est une maladie systémique idiopathique, associant une double symptomatologie cutanée et musculaire. La maladie fait partie de dermatose paranéoplasique. L’association de la dermatomyosite à un cancer rectal est rarement décrite dans la littérature. Nous présentons un cas d’une patiente atteinte de dermatomyosite paranéoplasique associé à un adénocarcinome rectal métastatique, et qui a présenté une symptomatologie clinique typique d’une dermatomyosite paranéoplasique, confirmée par les autres examens complémentaire (taux de créatine phosphokinase (CPK) + EMG (électromyogramme) + biopsie cutanée). La patiente était mise sous chimiothérapie, mais après sa deuxième cure, la patiente a présenté une aggravation rapide de son état général, décédée en quelque jours dans un tableau de défaillance multi-viscérale. Notre objectif est d’illustrer le caractère agressif de la dermatomyosite paranéoplasique et de faire une mise au point des connaissances actuelles concernant la place de la chimiothérapie dans la dermatomyosite néoplasique.

## Abstract (en)

Dermatomyositis is a systemic idiopathic disease characterized by a combination of both muscle and skin symptoms. It is a paraneoplastic dermatosis. Its association with rectal cancer has been rarely described in the literature. We here report the case of a female patient with paraneoplastic dermatomyositis associated with metastatic rectal adenocarcinoma presenting with clinical symptoms commonly found in subjects with paraneoplastic dermatomyositis. Other complementary examinations (CPK test + EMG + skin biopsy) were performed which confirmed this diagnosis. The patient underwent chemotherapy, but after the second cycle, she experienced a rapid worsening of her general condition and died after some days in a state of multisystem organ failure. This study aims to highlight paraneoplastic dermatomyositis’ aggressive nature and to update current knowledge on the importance of chemotherapy in the management of neoplastic dermatomyositis.

## Introduction

La dermatomyosite (DM) est une maladie systémique idiopathique, associant une double symptomatologie cutanée et musculaire. Le diagnostic est basé sur des critères qui sont toujours d’actualité. La dermatomyosite peut s'associer à des pathologies néoplasiques avec une fréquence d'environ 10 à 15% [1]. L’association d’une dermatomyosite à un cancer rectal est rarement décrite dans la littérature. Nous rapportons le cas d’une patiente admise en oncologie médicale pour prise en charge d’un cancer rectal révélé par une dermatomyosite.

## Patient et observation

**Figure 1:**
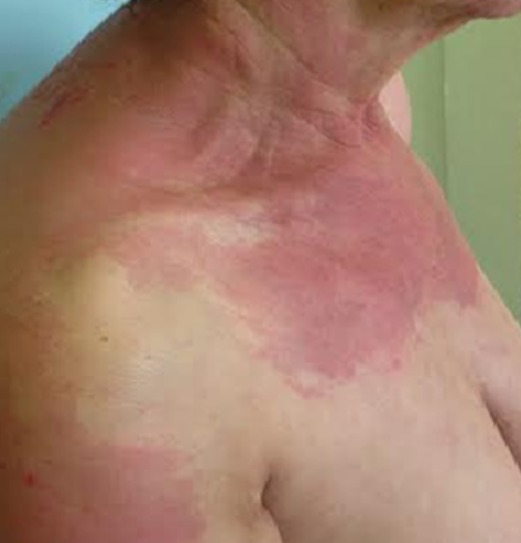
Vue de profil de la patiente montrant un placard érythémateux mal limité *

**Figure 2:**
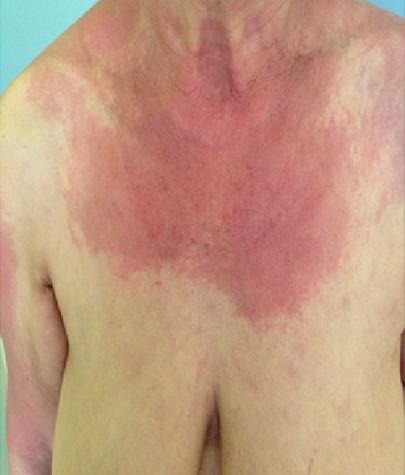
Image montrant un placard érythémateux mal limité à bordures émiettés par endroits et décolleté au niveau du cou et du bras

**Figure 3:**
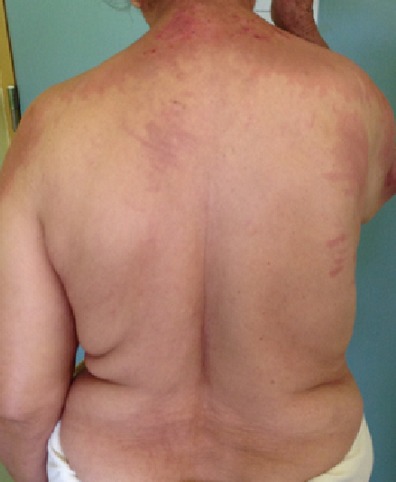
Placard érythémateux au niveau du dos, recouverts par des lésions de grattage et des excoriations avec un érythème flagellé

Il s’agit d’une patiente âgée de 74 ans, sans antécédents pathologiques, qui a présenté six mois avant sans admission des lésions cutanées prurigineuses, dont l’évolution était marquée par l’apparition d’une asthénie généralisée associé à un syndrome rectale motivant sa consultation. L’examen clinique a révélé la présence des lésions cutanées fait d’un placard érythémateux mal limité à bordures émiettés par endroits et décolleté (signe de châle +) au niveau du cou, les deux bras et le dos, recouverts par des lésions de grattage et des excoriations avec un érythème flagellé surtout au niveau du dos (Figure 1, Figure 2, Figure 3). Associé à un déficit musculaire prédominant au niveau proximal. Le reste de l’examen a objectivé une hépatomégalie dure, une masse franchissable au toucher rectal avec du méléna. Les investigations (biologiques, radiologiques, anatomopathologiques) ont objectivé le diagnostic d’un adénocarcinome rectal métastatique au niveau hépatique non résécable, associé à une dermatomyosite paranéoplasique qui était suspectée initialement, et confirmée par l’association d’une perturbation du bilan biologique (LDH à 1280 Ui/l, CPK à 5107 UI/l, CPKmb: 307 IU/l, l’anomalie à l’électromyogramme (EMG) qui a objectivé un tracé myogène, et la biopsie cutanée et musculaire qui sont revenu en faveur d’une dermatomyosite. Le dossier est discuté en réunion de concertation multidisciplinaire. La décision était une chimiothérapie palliative. Après sa première cure de chimiothérapie (protocole FOLFOX4) associée à une corticothérapie orale forte dose, la patiente a présenté de façon rapide une dégradation de son état générale, décédée dans un contexte au service des urgences dans un tableau de défaillance multiviscérale.

## Discussion

La dermatomyosite est une myopathie inflammatoire primitive d’étiologie inconnue, associant une atteinte cutanée avec une atteinte inflammatoire des muscles striés et prédominant aux ceintures [1]. La pathogénie de cette myopathie est encore mal comprise [2]. Les manifestations cutanées de la dermatomyosite associent rash héliotrope et papule de Gottron. Le rash héliotrope, avec ou sans œdème, est caractéristique dans sa localisation périorbitaire. Les papules sont localisées en regard des petites articulations des mains au niveau des coudes, des genoux ou des pieds. Un érythème péri-unguéal avec télangiectasies est assez caractéristique sans être pathognomonique. Des localisations au cuir chevelu sont habituelles. Les manifestations cutanées de la dermatomyosite précèdent souvent l'atteinte musculaire et peuvent persister alors que la myopathie est en rémission. L’atteinte musculaire est à l'origine d'une fatigabilité musculaire et parfois de myalgies. La présence d'une dysphagie proximale est le reflet de l'atteinte de la musculature striée du pharynx ou de l'œsophage supérieur. D’autres manifestations systémiques sont possibles: atteinte pulmonaire (essentiellement la pneumopathie interstitielle et le syndrome restrictif par hypoventilation), arthralgies ou arthrite, manifestations cardiaques, vascularite ou carcinose particulièrement chez l'enfant et l'adolescent atteint de dermatomyosite [2,3].

En 1975, Bohan et Peter ont proposé des critères diagnostiques de la dermatomyosite, dont elle est considérée comme certaine si les cinq paramètres suivants sont présents, et elle est probable si les seuls 4 premiers critères sont trouvés [4]: déficit moteur proximal, épargnant les muscles faciaux et oculaires, d’évolution rapidement progressive; tracé myogène à l’électromyogramme; élévation des enzymes musculaires; histologie musculaire décelant des infiltrats inflammatoires péri-fasciculaires, périmysiaux ou péri vasculaires, et une atrophie péri-fasciculaire; existence d’un rash cutané et/ou d’une carcinose. De même, Mastaglia *et al.* ont également proposé en 2002 des critères diagnostiques au cours des PM et des DM, en incluant, d’une part, les manifestations cliniques extra-musculaires de ces affections, et d’autre part, en affinant les résultats fournis par l’histologie musculaire au cours des dermatomyosites [5]. La dermatomyosite peut être associée à des pathologies néoplasiques avec une fréquence d'environ 10 à 15%, cette association à une corrélation étroite avec l'âge: plus de 50% des patients âgés de 65 ans ont un cancer associé [1]. Les publications des cas de dermatomyosite associé à un cancer remontent au début du siècle, et pourtant la réalité de la dermatomyosite paranéoplasique est restée longtemps discutée [6]. Deux premières études portant sur de grandes cohortes de patients, respectivement de 788 [7] et 539 patients [8] ainsi qu'une méta-analyse regroupant 1 018 patients [9] confirment de façon certaine l’augmentation du risque de cancer au cours des dermatomyosites.

Les cancers les plus fréquemment retrouvés associés aux dermatomyosites dont le risque est au moins trois fois supérieur à celui de la population générale sont les adénocarcinomes comme les cancers de l’estomac, du pancréas, du poumon et des voies respiratoires, de l’ovaire et les lymphomes [10]. L’association d’une tumeur rectal à une dermatomyosite est très rare, un nombre limité des cas ont étaient publiés dans la littérature [7, 11-13]. Le traitement spécifique de la dermatomyosite paranéoplasique permet d’améliorer le taux de survie [14]; il est basé essentiellement sur le traitement étiologique de la tumeur primitive, associé à une corticothérapie qui permet de diminuer la gravité des symptômes [11, 12]. Toutefois, dans quelques cas rapportés dans la littérature, le traitement du cancer peut apporter la guérison de la dermatomyosite [15]. Le pronostic de cette affection est souvent péjoratif, il dépend de plusieurs facteurs tels que: l'âge du patient, la sévérité de la myosite, la présence de la dysphagie, l'implication cardiaque, le diagnostic précoce et le type du traitement, en plus de la réponse du patient au traitement avec corticostéroïdes [16].

## Conclusion

Nous rapportons un cas de dermatomyosite paranéoplasique associé à un adénocarcinome rectal métastatique, afin d’apprécier la gravité de cette association qui peut mettre en jeu le pronostic vital, dont le traitement étiologique reste la base de la prise en charge thérapeutique.

## Conflits d’intérêts

Les auteurs ne déclarent aucun conflit d'intérêts.
